# Idiopathic granulomatous mastitis: a mimicking disease in a pregnant woman: a case report

**DOI:** 10.1186/1756-0500-6-95

**Published:** 2013-03-14

**Authors:** Juan A Garcia-Rodiguez, Andrew Pattullo

**Affiliations:** 1 Department of Family Medicine, University of Calgary, Sunridge Family Medicine Teaching Centre, 2685 – 36 Street NE, Calgary, AB, T1Y 5S3, Canada; 2 Department of Internal Medicine, Division Infectious Diseases, Rockyview General Hospital, 7007 14th Street SW, Calgary, AB, T2V 1P9, Canada

**Keywords:** Granulomatous mastitis, Breast abscess, Idiopathic, Diagnosis, Corticosteroid

## Abstract

**Background:**

Idiopathic granulomatous mastitis is a rare, benign, inflammatory chronic condition of unclear etiology.

This case is reported because it illustrates how idiopathic granulomatous mastitis can mimic other diseases, making it difficult to associate the presenting symptoms and the correct diagnosis; This disease is a challenge for clinicians to diagnose, manage and avoid iatrogenic complications, and requires consultation with experts in several specialties.

**Case presentation:**

The patient was 30 years old, South-American, eleven weeks pregnant, and with an apparent infectious mastitis. She presented with progressive worsening of her breast symptoms and multiple negative laboratory tests. She suffered different side effects from several prescribed treatments and endured a prolonged recovery. The article emphasizes the need for ruling out common pathologies to arrive at the correct diagnosis such as bacterial and fungal infections; granulomatous conditions like tuberculosis and sarcoidosis; and inflammatory breast carcinoma. It also describes frequently used pharmacological and supplementary forms of treatment for patients with this condition.

**Conclusion:**

Idiopathic granulomatous mastitis is a rare unusual condition of unknown etiology. Pathological confirmation is required for its diagnosis and optimal management is still unclear. The presentation and management of this case is intended to advance its awareness to physicians from different specialties.

## Background

Idiopathic granulomatous mastitis (IGM) is an uncommon, benign, inflammatory chronic condition of unclear etiology [1]. A 2011 review article reported 541 cases since 1972 [2]. It is also known as granular lobular mastitis and usually poses a diagnostic and therapeutic dilemma [3]. It is a relatively recent reported condition that was first described in 1972 by Kessler and Wollock [4]. The primary presentation is a firm breast mass, frequently associated with local pain and eventually skin ulcerations, abscesses and fistulae develop [3,5]. The clinical presentation frequently mimics breast abscess, infective mastitis and breast cancer [6]. Histopathological study is required to make the diagnosis, and findings include noncaseating granulomatous lobulitis making it imperative to rule out other granulomatous diseases.

Definitive treatment has not yet been defined [7]. Treatment most frequently includes corticosteroids [8] and immunosuppressants [2], although colchicine has been used [3]. Management can sometimes require drastic measures such as mastectomy [1,9].

Review articles aim to clarify the diagnosis and management [2,5,7,9] and most authors debate the suitability of either conservative or surgical treatment [1].

## Case presentation

A 30-year-old Colombian woman, G3,P1,A1, who was in her 11th week of an uneventful pregnancy, complained of one week of severe breast pain and had noted a small breast lump. She reported no signs of infection. She had breast fed her last baby eight months earlier. Her medical and surgical history was unremarkable, with no history of tuberculosis exposure and she had received tuberculosis vaccination (BCG) as a child. She had not taken contraceptives. Her family history was negative for breast cancer.

On physical examination a 3 cm. tender mass was palpated over the superior-external quadrant of her left breast with mild erythema. Her temperature was normal. A breast ultrasound was ordered.

Five days later her mass had grown to 9 cm. and was extremely tender to palpation; She was febrile (37.7°C). She was started on cephalexin and acetaminophen. On the 10th day, the ultrasound reported mastitis, with a macrolobulated focus and an area of possible fat necrosis around it. Her mass was now 12 cm. and a second 5 cm. mass had appeared.

She was urgently referred for drainage, which was attempted transcutaneously and was unsuccessful. Her pain became intolerable and during the following one and a half months she required two hospital admissions. Consultation and management were provided by physicians in internal medicine, emergency medicine, general surgery, gynecology, infectious diseases (including tuberculosis specialists), pathology, radiology and initial diagnoses made by these consultants included mastitis, breast abscess, breast carcinoma and tuberculous mastitis.

**Figure 1:**
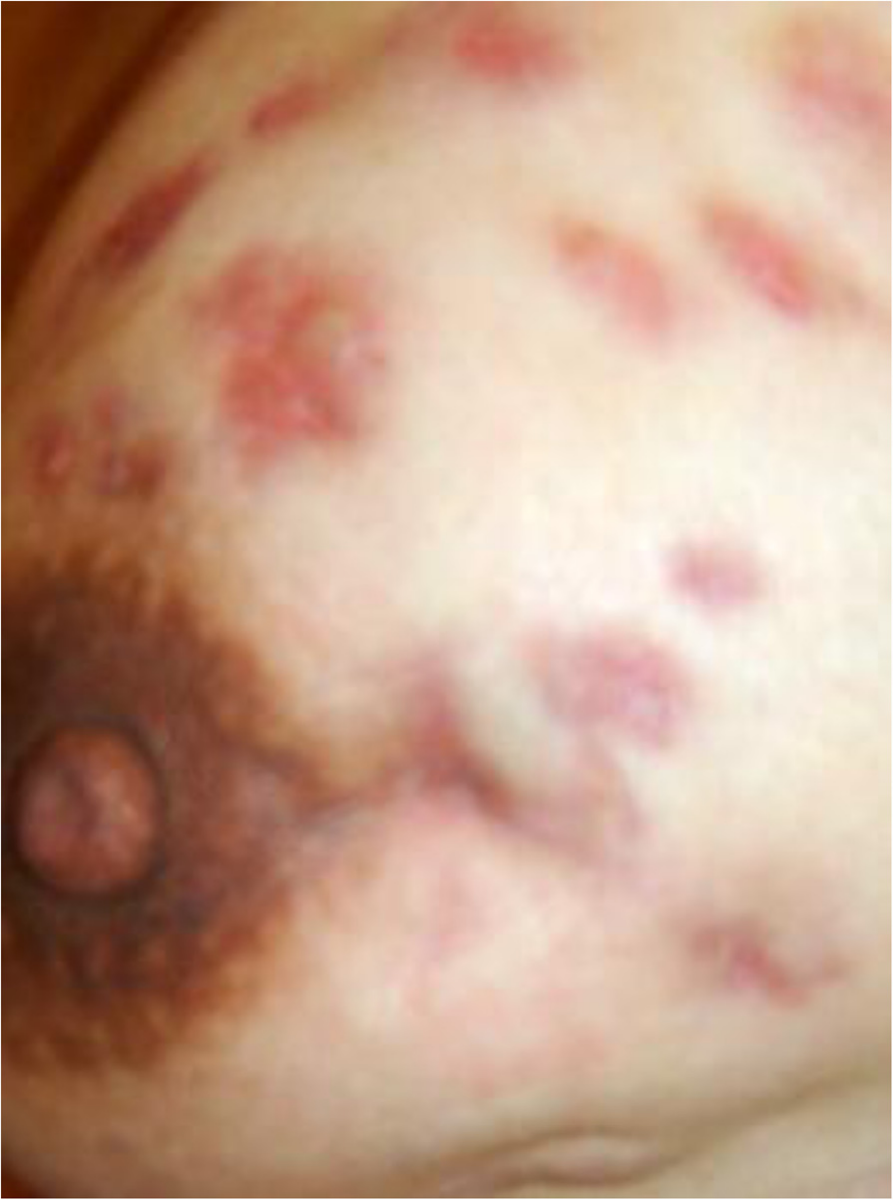
**A photograph of the patient’s left breast.** Clinical appearance of the patient’s breast including skin ulcerations, abscesses and fistulae. Photo obtained at the end of her first hospital admission.

**Figure 2:**
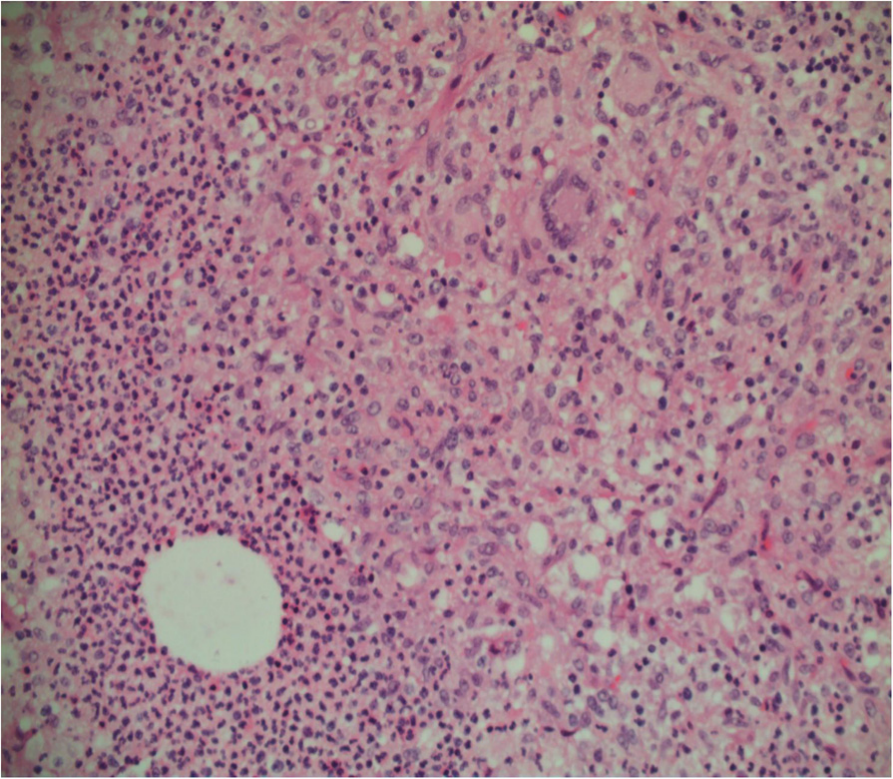
**Histology slide 1 of the patient’s left breast specimen.** Granulomatous inflammation with microabscess is observed, showing a granuloma with two giant cells and central inflammation. This sample was obtained by true-cut biopsy during her first hospital admission.

**Figure 3:**
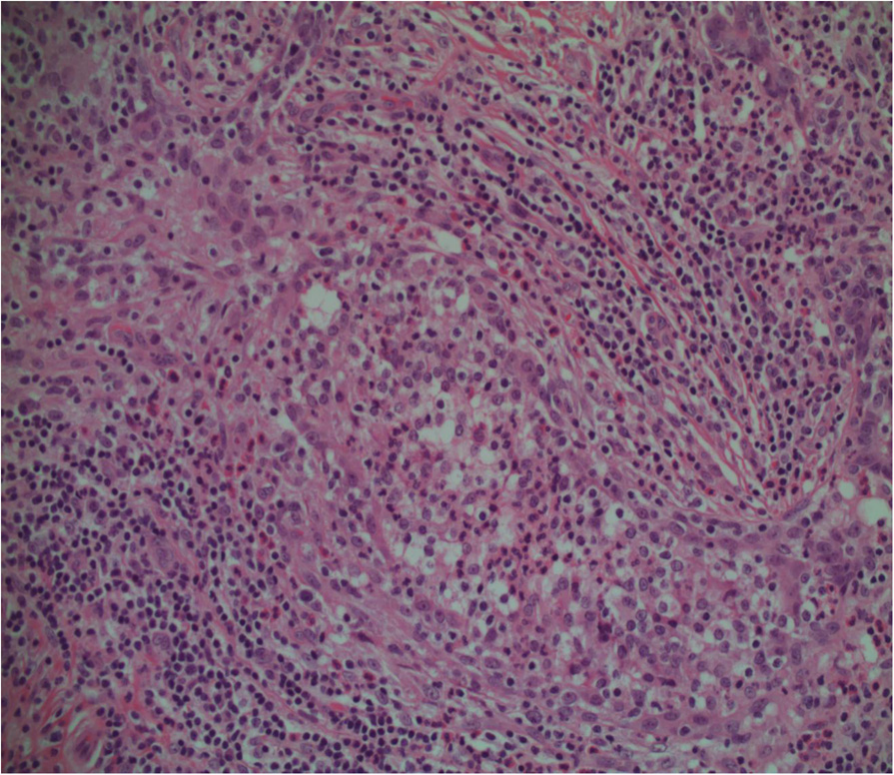
**Histology slide 2 of the patient’s left breast specimen.** Acute mastitis with polymorphonuclear cells within a terminal duct lobular unit.

Cultures of breast tissue initially reported scant *Haemophilus influenzae* and *Streptococcus viridians,* and the patient was treated sequentially with clindamycin, cefazolin, vancomycin, piperacillin-tazobactam combination, ceftriaxone and metronidazole with no improvement, and narcotics were required to control her pain. After the first dose of vancomycin the patient developed drowsiness, shortness of breath, a generalized rash and fever of 40.8 C degrees. She was diagnosed of having an atypical “red man” syndrome, from which she recovered uneventfully. She also had an allergic reaction to clindamycin. Chest radiographs demonstrated a calcified left upper lobe pulmonary granuloma. At the end of that month, incision and drainage was performed twice, with multiple biopsy samples obtained. Further bacterial cultures, fungal cultures and tuberculosis cultures and stains were negative. While undergoing these treatments the breast lesions progressed to involve most of the left breast, skin fistulae formed, thick material exuded from the surgical drains (Figure 1) and the patient remained febrile. The pathology reports noted granulomatous inflammation with plasma cells, giant cells, macrophages, lymphocytes and a single granuloma with central necrosis, and ruled out breast carcinoma (Figures 2 and 3). At the end of her hospitalization period the final diagnosis was IGM, the patient was started on prednisone 20 mg. four times daily, and some improvement of symptoms occurred. This medication dose varied from 60 mg. four times daily to 15 mg. daily based on her evolution.

She continued her pregnancy, the symptoms were more tolerable, and she had a normal vaginal delivery at 40 weeks. She breast-fed her baby with her right breast for two months. She continued prednisone treatment on the same variable doses. Her left breast continued to have scattered areas of drainage and new areas of tenderness developed.

One month after her delivery treatment options were re-considered by a multidisciplinary team and due to the deterioration in her symptoms a mastectomy was recommended. The patient disagreed with the decision, and returned to Colombia to obtain further opinions.

In Colombia she was treated with minocycline for six months and two 10 day cycles of IV amikacin. Deflazacort was given to control local inflammatory relapses. Triamcinolone and amikacin injections were given to the inflammatory nodules. The discharge from her lesions stopped six months after starting this treatment and the induration and redness disappeared one month later. She returned to Canada and continued taking only deflazacort, varying its dose depending on the presence of side effects. One year later, her symptoms and lesions had been controlled but, decreasing her deflazacort dose led to reappearance of discomfort and redness. The patient maintained her 6 mg. dose for four months remaining asymptomatic, at which time, she decided to stop her medication completely. Her lesions never returned and she has been asymptomatic until now.

## Discussion

This IGM case demonstrates potential difficulties in diagnosis and management, iatrogenic complications, the need to involve different areas of expertise, and the absence of knowledge about the etiology of IGM.

IGM is a relatively recent reported condition which was first described in 1972 by Kessler and Wollock [4]. It has also been called granular lobular mastitis by Going et al since 1987, based on histological samples that had a lobule-centered distribution from patients with the disease [10]. It is a rare, benign, inflammatory chronic condition of unclear etiology. The primary presentation is a firm breast mass, which is tender. It has been reported that up to 25% of cases can involve both breasts [1]. The full presentation includes breast masses, tumorous indurations, skin ulcerations, inflammation, local pain, tenderness, galactorrhea, abscesses and fistulae. Some authors have suggested that IGM is a self-limiting condition [1], with a range of two to 24 months [9] but a chronic presentation could last for several years.

IGM is seen more frequently in premenopausal women, but the age of presentation can widely vary. It has been reported in an 11 year old girl [7] as well as cases in the 6th, 7th and 8th decades [6]. Associations have been suggested with breast feeding, use of contraceptives, autoimmune disorders, alpha-1 antitrypsin deficiency and hyperprolactinemia [5,8]. Diagnostic tools such as ultrasound, mammogram, true-cut biopsy, fine needle aspiration biopsy and contrast-enhanced MRI can be helpful in diagnosis. A true-cut biopsy is recommended to clarify the diagnosis [11]. Other entities such us malignancy, fungal, bacterial and parasitic infections, autoimmune conditions, and other granulomatous diseases such as tuberculosis and sarcoidosis should be excluded. The diagnostic histopathologic changes include chronic granulomatous lobulitis without caseating necrosis plus giant cells, leucocytes, epitheloid cells, macrophages and micro-abscesses.

IGM is usually a diagnostic and therapeutic dilemma, which initially can mimic other conditions and is a diagnosis of exclusion. The clinical presentation frequently suggests breast abscess or breast cancer [9,12]. Some authors have mentioned that more than 50% of cases can mimic breast carcinoma [7]. The patient reported in this article started her clinical picture with unspecific findings of an expanding mass and surrounding local inflammation, as is usually described [5,8]. As with most patients with IGM the initial presentation leads to the use of antibiotics. In this patient, symptoms developed to suggest an infective mastitis or possibly an abscess. As her condition worsened, empiric antibiotic treatment was started.

When the diagnosis suggests mastitis, the lack of response in patients suffering from this condition can lead to different antibiotic combinations and the uncertain treatment could lead to complications, as was the case of this patient (“Red man” syndrome and allergic reaction to Clindamycin).

As the study of patients with this clinical presentation requires exclusion of other causes of mastitis and malignancy, obtaining core specimens by true-cut biopsy for pathological study is paramount. In this case, the pathology ruled out neoplasms but reported abscesses with positive bacterial cultures leading to antibiotic treatments. However, the presence of a granuloma and giant cells suggested IGM. Having a granuloma with central necrosis and evidence of a calcified pulmonary nodule on x-ray made tuberculosis a possible etiology, but the different cultures and stains were always negative for tuberculosis and her previous history was noncontributory for this diagnosis.

In the case presented, the patient emphatically refused to accept the radical surgical treatment that was proposed to her, her tenacity made her maintain her wish for conservative management, and her desperation lead her to look for further treatment in Colombia that helped to stabilize her situation. There is still debate about the treatment for this condition but it should initially be non-operative [1,5]. Conservative treatment with close observation has been suggested. In most of the cases once infectious conditions have been excluded oral steroid therapy is required [1,8]. Most of the evidence that supports these interventions comes from original articles and case reports. Anti-inflammatory medications, systemic antibiotics, methotrexate, colchicine, local infiltrations and ulcers wound care are also helpful. One study documented relapse in 50% of cases after discontinuing corticosteroid treatment. In those refractory to treatment or which recur, methotrexate or azathioprine are recommended [2]. In more difficult cases immunosuppressive management and complete surgical excision can be considered [6].

## Conclusions

A high index of suspicion is required to make the diagnosis the diagnosis of IGM. During the diagnostic process is important to rule out invasive carcinoma, breast infections and other granulomatous conditions. Treatment will remain a challenge in the absence of more specific disease understanding and optimal decisions for care will often draw on the judgment of clinicians from many areas of expertise.

## Competing interest

The authors declare that they have no competing interests.

## Authors’ contributions

JAGR is the primary health provider for the patient, conceived, designed, compiled the data for the article and wrote the article. AP is also a treating physician for the patient, contributed for the editing and design of the article. Both authors gave final approval for its publication.

## Authors’ information

JAGR is the primary health provider for the patient, who practices Family Medicine and Sports Medicine. He is an Assistant Professor at the Family Medicine Department of the University of Calgary.

AP is an Infectious Diseases Specialist who provided medical care for the patient. He is a Clinical Associate Professor working at the Rocky View General Hospital in Calgary.
